# Effect of Maxillary Lateral Incisor Inclination on Attractiveness Perception of Smile Esthetics among Orthodontists and Laypersons in Close Up and Full Face View: Effect of Maxillary Lateral Incisor Inclination on Attractiveness Perception

**DOI:** 10.31661/gmj.v13iSP1.3602

**Published:** 2024-12-25

**Authors:** Fatemeh Moghimi, Abbas Salehi Vaizri, Atefe Ahmadvand

**Affiliations:** ^1^ Faculty of Dentistry, Shahed University, Tehran, Iran; ^2^ Department of Orthodontics, Faculty of Dentistry, Shahed University, Tehran, Iran; ^3^ Department of Orthodontics, School of Dentistry, Shahid Beheshti University of Medical Sciences, Tehran, Iran

**Keywords:** Orthodontists, Malocclusion, Face, Esthetics, Dental

## Abstract

**Background:**

Maxillary lateral incisors are crucial in contributing to a pleasing smile. As earlier research has not examined how the labiopalatal inclination of maxillary lateral incisors influences smile appeal, this study sought to investigate this factor from the viewpoints of both laypeople and orthodontists; it also evaluated whether a close-up or full-face view of the smile and the model’s gender influence the attractiveness of the smile.

**Materials and Methods:**

Three-dimensional dental models and images of a male and female volunteer were created and subsequently modified to display seven labiopalatal incilination values (-15°, -10°, -5°, 0°, +5°, +10°, and +15°). These were assessed by both orthodontists and laypeople from two perspectives (close-up and full-face) through an online survey.

**Results:**

The analysis revealed no significant differences between the full-face and close-up perspectives. However, the male participant with +15° and +10° inclinations demonstrated a noticeably higher level of attractiveness in the full-face view. Laypeople assessed smiles with inclinations of +10° and +15° as more attractive, whereas orthodontists found 0° to be more appealing. Both groups agreed that the inclination of 0° was the most attractive. Conversely, the least appealing smile was considered to be -15° by laypeople, and +15° by the orthodontists.

**Conclusion:**

The palatal inclination of the maxillary lateral incisors was deemed more acceptable than labial torque. Furthermore, orthodontists demonstrated a greater level of rigor in their evaluation of the attractiveness of smiles.

## Introduction

An attractive smile affects life and self-confidence [1]. One of the principal objectives of orthodontic treatments is to create an appealing smile [2]. Modern dentistry should focus on improving smiles by managing teeth and soft tissue. This can involve moving teeth, modifying soft tissue, performing restorative procedures, or using a combination of them [3]. Maxillary lateral incisors can influence the smile's attractiveness due to their position in the smile arch [4]. Earlier research has examined how the incisal edge height, the position from front to back, the length of the incisogingival area, the level of the gingival margin, deviation from the golden ratio, and the angle of the maxillary lateral incisors influence the attractiveness of a smile [4][5][6][7]. Haerian et al. conducted a study to evaluate the perception of smile attractiveness among orthodontists, general dentists, and laypersons [5]. The results revealed a unanimous preference across all groups for the lateral incisor to exhibit a gingival height positioned 1 mm more incisally than that of the central incisor, with the incisal edge of the lateral incisor positioned 1 mm more apically than the central incisor for optimal aesthetic appeal [5]. Among orthodontic specialists, a mesial angulation of 20 degrees received the highest attractiveness ratings, while general dentists and laypersons favored mesial angulations of 10 and 15 degrees, respectively [5].

Taki et al. investigated the views on smile photographs held by orthodontists, laypeople, and general dental practitioners [7]. Orthodontists favored the golden proportion (62%-67%), while both general dental practitioners and laypeople chose 67% as their preference. Regarding gingival display, all three groups highly rated the corrected heights of −0.5 and −1 mm [7]. For the length of lateral incisors, laypeople preferred −0.5 mm, whereas orthodontists and general dental practitioners opted for −1 mm [7].

Jiang et al. conducted a study on the perception of smile attractiveness among laypeople and orthodontists [4]. Their findings revealed that orthodontists rated a smile adjusted to +1.5 mm as the least attractive, while laypersons deemed both +1.5 mm and -1.5 mm smiles as the least appealing. In contrast, all participants agreed that a smile at 0 mm was the most attractive [4].

Karaahmetoğlu et al. examined how doctoral students and dental technicians perceive the aesthetics of maxillary lateral incisors by altering the widths (52%, 62%, 72%) and reducing the incisal edge lengths (0.5, 1, 1.5 mm) in photographs of ideal smiles [8]. For male images, width ratios and edge lengths had no significant effect on aesthetic perception (p≥0.05) [8]. However, in female images, although overall evaluations showed no significant difference (p≥0.05), width ratios and edge lengths did have a significant impact (p≤0.05) [8]. Participants identified the most aesthetically pleasing combination as a 62% width ratio and a 0.5 mm reduction in incisal edge lengths [8].

Lavanya et al. conducted research to evaluate the golden proportion, golden mean, and Preston proportion of six maxillary anterior teeth [9]. Their findings indicated that there were no significant differences in the golden proportion and golden mean based on gender, whereas the Preston proportion did show statistical differences in the overall population [9]. The study concluded that Ward's formulas for the golden mean and golden proportion are effective tools for smile design and full-mouth rehabilitation [9].

Dag et al. investigated how various esthetic dental proportions, created using a digital smile program, affected perceptions among different demographic groups [10]. They evaluated four types of dental proportions (golden proportion, golden percentage, Preston proportion, and recurring esthetic dental proportion) along with two tooth shapes (oval and square) as assessed by dental students, dentists, and non-professionals [10]. The findings showed that gender did not influence the ratings (P>0.05), but participants aged over 30 and non-professionals gave higher scores to the designs (P<0.05) [10]. When factoring in tooth shape and gender, the GPR design was rated lower than the RED design (P<0.001) [10].

Alveolar bone limits the spatial movement of the tooth and uncontrolled movement of the tooth causes different problems like dehiscence, fenestration, and external root resorption [11]. These incidents are more likely to occur in the apical area of maxillary lateral roots due to the presence of a depression called the "Lateral Fossa" in the alveolar bone [11][12]. Orthodontists can plan their treatment according to the range of acceptable labiolingual inclinations for laypeople to achieve aesthetic goals and also maintain periodontal health.

However, factors such as differences in skin tone, the application of lipstick, and tooth shape also impact smile attractiveness. Some authors suggest including a full-face view to show how all smile-related features interact [13][14].

Prior studies have not explored how the labiopalatal inclination of the maxillary lateral incisors influences the perception of smile attractiveness. As a result, the originality of this study lies in examining how different labiopalatal inclinations of the maxillary lateral incisors affect smile appeal. The research utilized advanced methods, such as 3D and intraoral scanning techniques, while combining both 3D and 2D analyses to obtain thorough findings. This study aimed to investigate the impact of maxillary lateral incisor inclination on smile attractiveness in laypeople and orthodontists and explore how a close-up or full-face smile and the gender of the smile model can influence smile attractiveness.

## Methods and Materials

This cross-sectional study received approval from the research ethics committee with control number IR.SHAHED.REC.1401.183.

### Acquisition of 3D Dental Models and Photographs

A 23-year-old male and a 20-year-old female volunteer who had not undergone any aesthetic or orthodontic treatments were chosen as smile models. The smiles were evaluated as very attractive. The selection criteria included the following: well-aligned teeth, convex and consonant smile arch, symmetric maxillary incisors, maximum gingival display up to 2 mm, fully maxillary incisors display, buccal corridor with average width, 0.5 mm distance between the incisal edge of the lateral and central incisors, 0-0.5 mm distance between the gingival margin of lateral incisors and adjacent teeth, normal overjet and overbite [15][16]. The facial criteria included matching dental and facial midlines, apparent facial symmetry in the frontal plane, bilateral symmetry in the fifths of the face, and the interpupillary line parallel with the true horizontal plane in the NHP (Natural Head Position) [17][18].

Photographing the full face and close-up view of the smiles was done with a camera (D7500; Nikon, Tokyo, Japan) under the following conditions: the camera was at the same height as the smile, parallel to the horizon, 1 meter away from the smile for full-face imaging, and 0.5 meters away from the smile for close-up imaging. The volunteers stood while the interpupillary line was parallel with the true horizontal plane in NHP.

Before photographing, the social smile was practiced by the volunteers. Photographing of both volunteers was done in the same situation and light conditions. Digital three-dimensional (3D) models of the maxillary teeth of both volunteers were obtained in the same lighting and environmental conditions with an intraoral scanner (iATON intraoral scanner, Milan, Italy) and saved in STL format.

### Editing 3D Dental Models

We used Autodesk Meshmixer software (Autodesk Inc.: Version 3.5) to edit the 3D models. The following steps were taken: 1. Mirrored transformation of the dental arch from the right side 2. Size calibration to ensure that the incisogingival height of the maxillary right central incisors of both 3D models matched the clinical view. 3. Measured and recorded the labiopalatal inclinations of the lateral incisors (the angle between the longitudinal axis of the tooth and the horizontal plane). The three-dimensional coordinates of the crown centers on the labial surface, also known as the Incisor Facial Axis Point (according to Andrews) [19], were also recorded. This point is located between the incisal and gingival on the midsagittal plane of the crown's labial surface [19].

We used the labiopalatal inclinations of the right lateral incisors of both models as the standard labiolingual inclination (0°). In Autodesk Meshmixer software, we utilized the transform tool to modify the labiolingual inclinations of the lateral incisors on both sides. We made labiolingual inclination adjustments while maintaining the original anterior-posterior position of the Incisor Facial Axis Points of the teeth, with a difference of five degrees: -15°, -10°, -5°, 0°, +5°, +10°, and +15° (- indicates palatal inclination, and + indicates labial inclination). After each adjustment, we captured a screenshot of the frontal view of the 3D models as a reference for the subsequent steps.

### Preparation of Photographs

The photographs of smiling models were edited using Adobe Photoshop software (Adobe, San Jose, Calif). The brightness, contrast, color, and background image whitening were adjusted as necessary. Subsequently, the right side of the smiles in close-up view images and the right side of the models' faces in full-face view images were mirrored.

**Figure-1:**
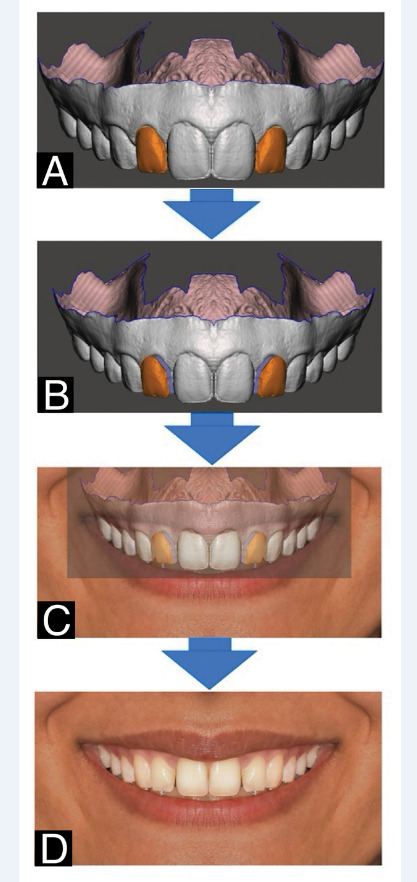
Figure 1. The steps of modifying the labiopalatal inclinations of the maxillary lateral incisors in photography: A) Selecting lateral incisors to change the torque. B) Taking the screenshot of the frontal view of the 3D dental model after applying changes (-10°). C) Overlapping the screenshot prepared with the original photography of the smile in Photoshop software. D) Creating the final torque change of the lateral incisors in photography.

To create consistent and accurate images, we used screenshots as editing templates in Photoshop. Each screenshot was overlaid as a new layer on the original image, with the opacity reduced to ensure the original smile was still visible. We aligned the two images by matching the incisal edge of the central incisors, the tips of the cusps of the canines and premolars, and the gingival margin of the canines and central incisors (Figure-1). Once the lateral incisors in the main smile had been fully selected, the transformation tool was used to move them to the position shown in the screenshot, ensuring a perfect match. The final image was obtained by deleting the screenshot layer. This process was repeated for male and female smile models, with all labiolingual inclination changes (Figure-1).

In total, we obtained 28 images, including:

1, 7 images of a female close-up smile with different labiopalatal inclinations for lateral incisors

**Figure-2:**
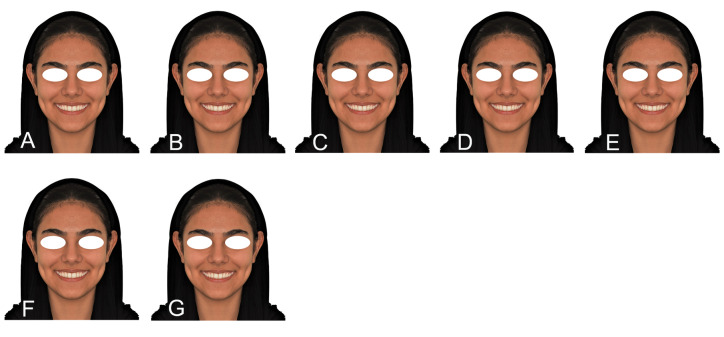
Figure 2. Torque changes of maxillary lateral incisors in the full-face smile of the female model. A, Lateral incisors with -15° torque B, Lateral incisors with -10° torque C, Lateral incisors with -5° torque D, Lateral incisors in control position (0°) E, Lateral incisors with +5° torque F, Lateral incisors with +10° torque G, Lateral incisors with +15° torque

**Figure-3:**
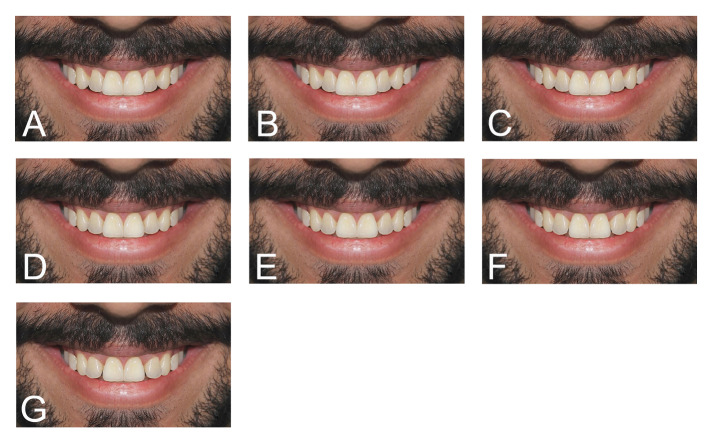
Figure 3. Torque changes of maxillary lateral incisors in the close-up smile of the male model. A, Lateral incisors with -15° torque B, Lateral incisors with -10° torque C, Lateral incisors with -5 ° torque D, Lateral incisors in control position (0°) E, Lateral incisors with +5° torque F, Lateral incisors with +10° torque G, Lateral incisors with +15°orque

2. 7 images of a female full-face smile with different labiopalatal inclinations for lateral incisors (Figure-2)

3. 7 images of a male close-up smile with different labiopalatal inclinations for lateral incisors (Figure-3)

4. 7 images of a male full-face smile with different labiopalatal inclinations for lateral incisors

### Evaluation of Photographs

The evaluation of the images was conducted by two groups: orthodontists and laypeople. The necessary sample size was determined using the GPOWER version 3.1.9.7 software, with a power analysis formula that considered a first type error of α = 5% and a test power (β-1) of 80%. As a result, the study required a sample size of 30 orthodontists and 30 laypeople.

An online questionnaire was utilized to assess the images. At the beginning of the questionnaire, participants received a brief overview of the research plan. After this introduction, pictures belonging to four distinct groups were shown in a random order, and each participant was asked to rate the attractiveness of each smile. A Likert scale from 1 (very unattractive) to 5 (very attractive) was employed for this purpose. The scale offered the following choices: 1 - Very unattractive; 2 - Unattractive; 3 - Neither unattractive nor attractive (neutral); 4 - Attractive; 5 - Very attractive. During the questionnaire, participants were allowed to go back to previous questions and change their answers, and there was no time limit for completing the questionnaire.

To assess reliability, 18 evaluators (9 orthodontists and 9 laypeople) were randomly asked to answer the same questions again after two weeks. Reliability was measured using the intraclass correlation coefficient (ICC) index.

### Statistical Analysis

The collected data was statistically analyzed and graphed using the free and open-source software R, version 4.2.1. Specialized packages were utilized, including dplyr, rstatix, and MASS, to perform statistical analysis. The Shapiro-Wilk test was used to measure the normality of the data, the Kruskal-Walli’s test was used to compare different labiolingual inclination values, and the Mann-Whitney test was used to compare two groups of evaluators and two views of smile. All graphs were created using R version 4.2.1.

## Results

We used the two-way random method of absolute agreement to determine the Intraclass Correlation Coefficient (ICC). The ICC was 0.84 (0.81-0.86) for both orthodontists and laypeople, indicating the high reliability of this study.

**Figure-4:**
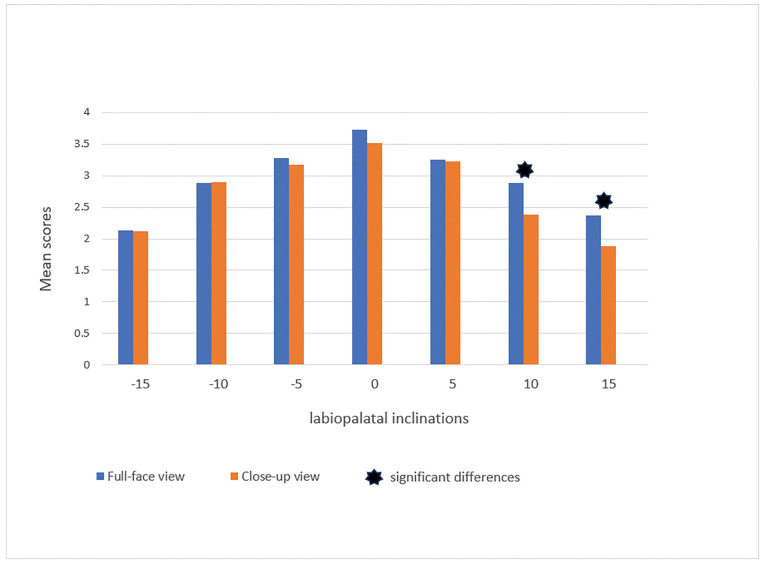
Figure 4. The average scores of perceptions of the attractiveness of the male smile in close-up and full-face views with different labiopalatal inclinations of maxillary lateral incisors.

**Figure-5:**
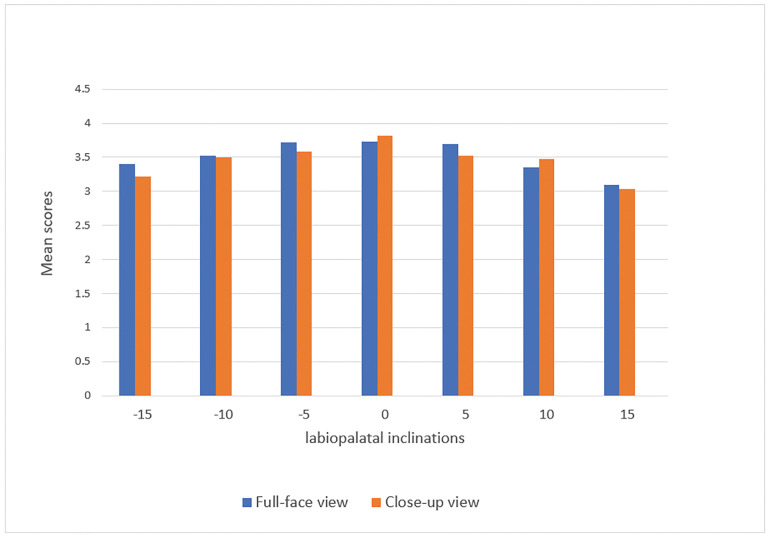
Figure 5. The average scores of perceptions of the attractiveness of the female smile in close-up and full-face views with different labiopalatal inclinations of maxillary lateral incisors.

We studied the effect of close-up and full-face smile views on smile attractiveness. This segment facilitated an evaluation of the impact that different facial features exert on the perception of smile attractiveness. The full-face view is more attractive than close-ups at inclinations of +10° and +15° in the male smiles (Figure-4). We found no significant difference in female smiles between the two views (P>0.05, Figure-5).

**Figure-6:**
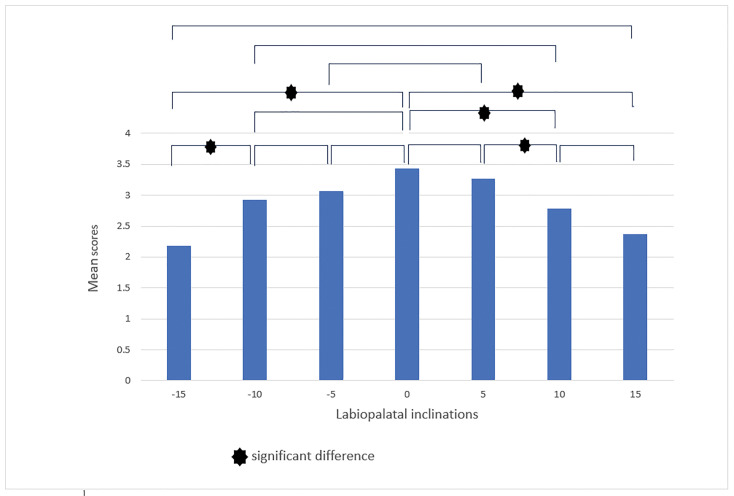
Figure 6. The pairwise comparison of male smiles with different maxillary lateral incisors torques, according to laypeople.

**Figure-7:**
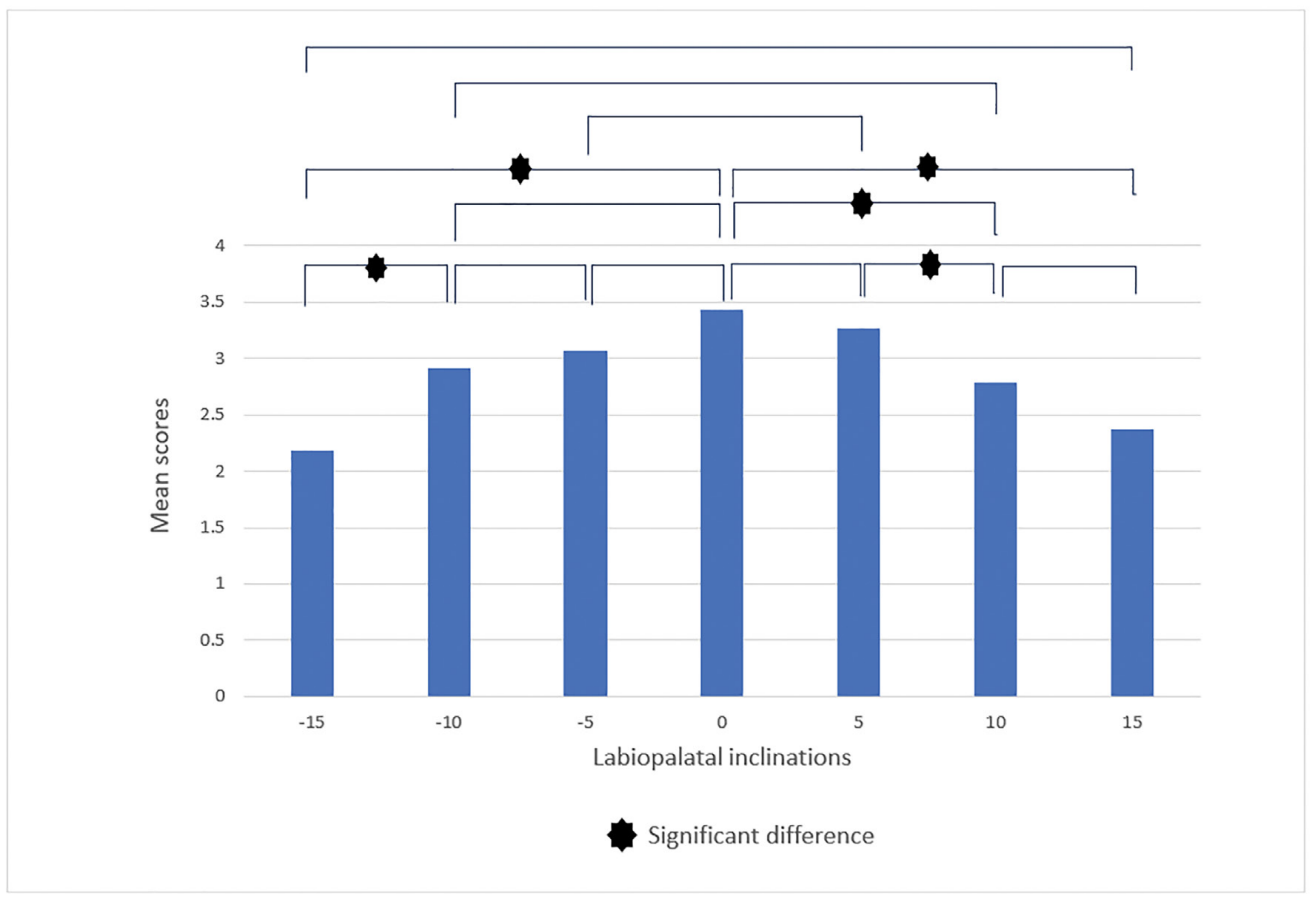
Figure 7. The pairwise comparison of male smiles with different maxillary lateral incisors torques, according to orthodontists.

**Figure-8:**
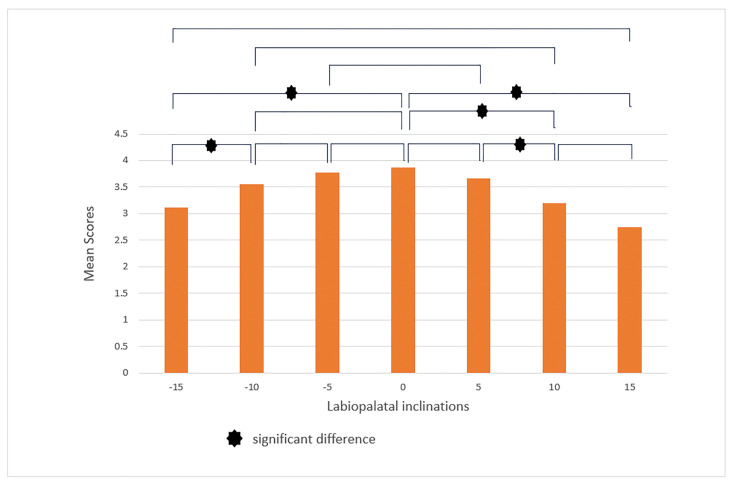
Figure 8. The pairwise comparison of female smiles with different maxillary lateral incisors torques, according to orthodontists.

**Figure-9:**
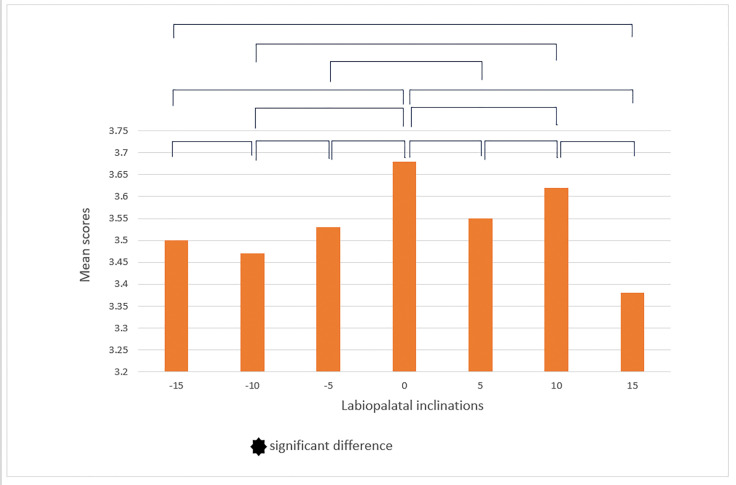
Figure 9. The pairwise comparison of female smiles with different maxillary lateral incisors torques, according to laypeople.

In the subsequent stage, we conducted a pairwise comparison of smiles with different labiolingual inclinations. The findings presented in this section offer insights into the evaluators' abilities to distinguish between five levels of differentiation and to respond with greater rigor accordingly. In the male smile, according to laypeople, the labiolingual inclinations (-10°, -15°), (0°, -15°), (0°, +10°), (0°, +15°), and (+5, +10) showed significant differences in terms of smile attractiveness (Figure-6). Orthodontists reported significant differences for labiolingual inclinations: (-10, -5), (0, -5), (0, +5), (0, -10), and (+15, +10) in addition to those previously mentioned (Figure-7). In the female smile, according to orthodontics, the labiolingual inclinations (-10, -15), (0, -15), (0, +10), (0, +15), and (+5, +10) showed significant differences (Figure-8). However, according to laypeople, there was no significant difference in smile attractiveness between the two different labiolingual inclinations (P>0.05, Figure-9). Then, we compared the opinions of two groups of evaluators. The results detailed in this section enable an examination of how changes in inclination affect the perception of smile attractiveness within two distinct groups of evaluators. However, one notable limitation of this method is the variation in gender and age among the evaluators, which could influence the accuracy of their evaluations. We discovered significant differences in the perception of male smiles between the two groups. We found that laypeople rated smiles with labiolingual inclination of +10° and +15° as more attractive, while orthodontists rated smiles with 0° labiolingual inclination change as more attractive (Table-1). Both groups agreed that the most attractive smile was one with 0°labiolingual inclination change (Table-1). On the other hand, the least attractive smile was determined to have a labiolingual inclination of -15°, according to the laypeople, and a labiolingual inclination of +15°, according to the orthodontists (Table-1).

There were noticeable differences in the female smile between orthodontists and laypeople in three labiolingual inclinations: -15°, +10°, and +15° (Table-2). Laypeople found the smile to be more attractive in these three labiolingual inclinations (Table-2). Both groups considered a 0° labiolingual inclination to be the most attractive, while a +15°labiolingual inclination was considered the least attractive smile (Table-2).

## Discussion

**Table:** Table1. Means and Standard Deviations of the Rating for the Male Smile Images by the Two Groups of Evaluators

**Variable**	**Laypeople**		**Orthodontics**		**P**
	Mean	SD	Mean	SD	
**-15**	2.18	0.96	2.07	0.66	0.68
**-10**	2.92	1.12	2.87	0.91	0.84
**-5**	3.07	1.12	3.40	0.87	0.083
**0**	3.43	0.93	3.82	0.81	0.017
**+5**	3.27	1.05	3.22	0.94	0.788
**+10**	2.78	0.96	2.48	0.95	0.033
**+15**	2.37	1.10	1.88	0.84	0.014

**Table:** Table2. Means and Standard Deviations of the Rating for the Female Smile Images by the Two Groups of Evaluators

**Variable**	**Laypeople**		**Orthodontics**		**P**
	Mean	SD	Mean	SD	
**-15**	3.50	0.87	3.12	0.92	0.022
**-10**	3.47	0.95	3.55	0.83	0.616
**-5**	3.53	0.95	3.77	0.74	0.130
**0**	3.68	0.95	3.87	0.89	0.29
**+5**	3.55	0.95	3.68	0.70	0.4
**+10**	3.62	1.00	3.20	0.88	0.015
**+15**	3.38	1.03	2.75	0.91	0.000

Effective torque control of incisors is crucial, especially in the area of the maxillary lateral incisors, due to the presence of a depression in the alveolar bone of the maxilla known as the lateral fossa [20]. Improper mechanical control of lateral incisor movement can result in root resorption, fenestration, and bone dehiscence [12]. Additionally, controlling the labiolingual inclination of maxillary lateral incisors is challenging using conventional bracket systems, particularly when adjacent teeth require opposing torque management [21]. However, the labiolingual inclination of maxillary lateral incisors has not been investigated in previous studies. Orthodontic treatment is designed to enhance both the aesthetic appearance and stability of occlusion. These two facets are essential for maintaining the health of the teeth and periodontium. Orthodontists must identify the optimal range of labiolingual inclinations of the anterior teeth that appeal to both patients and professionals. This range serves as a guide for developing a comprehensive treatment plan, ultimately ensuring the most favorable outcome for the patient. Additionally, it is critical to consider the influence of other facial features, including hair, nose, eyes, and eyebrows, on the overall appeal of a patient's smile [22]. Our study reveals that the perceived attractiveness of male smiles is significantly influenced by the viewing angle, specifically in close-up or full-face perspectives, when the maxillary lateral incisors exhibit labiolingual inclinations of +10° and +15°. In these specific inclinations, close-up views yield higher average attractiveness ratings. Conversely, the attractiveness of female smiles remains unaffected by the viewing angle (P>0.05) across all labiolingual inclination values of the maxillary lateral incisors. The results suggest that for male smiles, additional facial features—such as eyebrow shape, eye characteristics, and nose structure—play a vital role in influencing attractiveness when the maxillary lateral incisors have labial inclinations. In contrast, these facial attributes do not similarly impact the perceived attractiveness of female smiles when labiolingual inclinations of the maxillary lateral incisors are altered.

The findings of the study regarding female smiles are consistent with those of related research in the field [22][23][24]. For instance, Lemos et al. investigated the influence of various labiolingual inclinations of canines on the perceived attractiveness of a smile [23]. Their results indicated that there was no significant difference in the perception of smile beauty between orthodontists and laypersons, irrespective of whether the observations were made from a close-up or full-face perspective [23]. Similarly, Suzuki et al. assessed the effect of gingival display on smile attractiveness and concluded that this aspect did not lead to significant differences in perception between close-up and full-face views [22]. Furthermore, a study by Nascimento et al. analyzed the impact of different buccal corridor dimensions on the attractiveness of smiles, revealing that neither close-up nor full-face views significantly influenced the perception of smile beauty [24].

The findings regarding male smiles differ from those of previous studies [22][23][24]. These discrepancies may arise from factors such as the number of assessors, the study parameters, and the gender of the smiles being evaluated. Nonetheless, the results related to male smiles align with the research conducted by Flores-MIR [25]. In that study, Flores-MIR examined smile evaluations using three different types of images: full-face, close-up, and intraoral, as assessed by laypeople [25]. It was found that laypeople had a greater understanding of smile aesthetics in close-up views compared to full-face images [25].

Orthodontists noticed a difference of five degrees more than laypeople when comparing the labiolingual inclination of maxillary lateral incisors in pairs. This suggests that orthodontists are stricter in determining the attractiveness of a smile.

Our research has revealed notable disparities in the attractiveness evaluations of male and female smiles based on varying labiolingual inclinations, as perceived by orthodontists versus laypeople. Orthodontists assigned higher attractiveness ratings to the male smile exhibiting a 0° labiolingual inclination compared to laypersons. Conversely, laypeople demonstrated a preference for both male and female smiles with labiopalatal inclinations of +10° and +15°, as well as female smiles with a -15° inclination.

The findings indicate that orthodontists tend to prefer palatal inclination, whereas laypersons exhibit a mixed preference, favoring palatal inclinations at certain angles and labial inclinations at others. However, these preferences did not demonstrate statistically significant differences across the various cases studied. Additionally, the results emphasize that orthodontic specialists assess smile attractiveness with a more critical perspective compared to laypeople. The study's outcomes reveal that both groups share a similar perception of the female smile, while orthodontists' evaluations of the male smile align with previous research. For instance, Albwardi et al. identified that a labiolingual inclination of +15° for maxillary incisors in profile view was considered the least attractive smile among evaluators, and they found that lingual inclinations were generally more accepted than labial inclinations [26]. Cao et al. similarly found that incisors positioned upright or with a slight lingual inclination in profile view were perceived as the most attractive. They noted that incisors with a lingual tilt were considered more aesthetically pleasing than those with a labial tilt in profile view [4]. Lemos et al. observed that orthodontists rated smiles featuring maxillary canines with labiolingual inclinations of 0°, -5°, and -10° as the most appealing. In contrast, laypeople favored smiles with canines showing inclinations of 0°, -5°, -10°, -15°, and +5° as the most attractive [23]. Both groups concurred that smiles with labiolingual inclinations of +10° and +15° were deemed the least appealing [23]. Furthermore, the study revealed that changes in the lingual inclination of maxillary canines were perceived as more tolerable than changes in buccal inclination [23]. However, the findings regarding the perception of male smiles by laypeople contradict previous research [23].

The findings of this study may also serve interdisciplinary purposes. Agenesis of maxillary lateral incisors presents with varying prevalence across different ethnic groups [27]. Treatment options include space closure via orthodontic treatment and canine substitution, fixed prosthodontic bridges, temporary anchorage devices (TADs), and orthodontic space opening for single-unit implants [27][28][29]. Whether the approach involves cuspid substitution or the prosthetic replacement of the absent tooth, these cases often necessitate restorative compromises. For instance, in cuspid substitution, the crown may be excessively thick buccal-lingually, or the root inclination might be inadequate, leading to the appearance of the inclined tooth. This paper aims to assist the interdisciplinary team in evaluating the proposed outcomes and identifying the compromises that patients are willing to accept.

During our study, we faced limitations in translating 3D changes into 2D representations. Specifically, we observed that altering the labiolingual inclination of a tooth not only affects its position but also alters the brightness of various parts of the tooth in different ways. Implementing these changes in practical scenarios can influence the positioning of adjacent teeth as well as the gingival margin. Unfortunately, we were unable to apply these modifications within the scope of our study.

For future studies, we recommend assessing the perception of a smile's attractiveness by altering the position of the maxillary lateral incisors from various perspectives, including three-quarter and half-views. It would also be beneficial to evaluate the simultaneous impact of changing the positions of both adjacent teeth and the lateral incisors on the overall perception of the smile's attractiveness. Additionally, we plan to investigate the effects of modifying the position and labiolingual inclination of the lateral incisors in both unequal and unilateral manners on the perception of smile attractiveness in upcoming research.

## Conclusion

According to this study, evaluators determined that a 0° labiolingual inclination was the most aesthetically pleasing, while the palatal inclination of the maxillary lateral incisors was considered more acceptable than the labial inclination. Furthermore, there were no significant differences in the assessment of smile attractiveness between full-face and close-up views, with the exception of male smiles featuring +15° and +10° inclinations, which were found to be notably more attractive in a full-face context. Additionally, it was observed that orthodontists were more discerning in their evaluations of smile attractiveness compared to laypeople.The clinical significance of this study's results lies in the understanding that changing the inclination of lateral incisors can be challenging due to factors such as the presence of lateral fossa and the need to maintain periodontal health. Furthermore, due to the results of this study, there is a notable difference in how laypeople perceive the attractiveness of a smile compared to orthodontists. This less stringent perspective can provide a foundation for prioritizing treatment compromises over the pursuit of an ideal state tailored to individual cases. Therefore, it is essential to engage in meaningful discussions between the orthodontist and the patient to gain insight into the patient's perception of the ideal smile before commencing treatment.

## Conflict of Interest

None.
